# Comparison of public peak detection algorithms for MALDI mass spectrometry data analysis

**DOI:** 10.1186/1471-2105-10-4

**Published:** 2009-01-06

**Authors:** Chao Yang, Zengyou He, Weichuan Yu

**Affiliations:** 1 Laboratory for Bioinformatics and Computational Biology, Department of Electronic and Computer Engineering, The Hong Kong University of Science and Technology, Clear Water Bay, Hong Kong, PR China

## Abstract

**Background:**

In mass spectrometry (MS) based proteomic data analysis, peak detection is an essential step for subsequent analysis. Recently, there has been significant progress in the development of various peak detection algorithms. However, neither a comprehensive survey nor an experimental comparison of these algorithms is yet available. The main objective of this paper is to provide such a survey and to compare the performance of single spectrum based peak detection methods.

**Results:**

In general, we can decompose a peak detection procedure into three consequent parts: smoothing, baseline correction and peak finding. We first categorize existing peak detection algorithms according to the techniques used in different phases. Such a categorization reveals the differences and similarities among existing peak detection algorithms. Then, we choose five typical peak detection algorithms to conduct a comprehensive experimental study using both simulation data and real MALDI MS data.

**Conclusion:**

The results of comparison show that the continuous wavelet-based algorithm provides the best average performance.

## Background

Proteome research requires the analysis of large-volume protein data in a high-throughput manner. Mass Spectrometry (MS) is a common analytical tool in proteome research. It can be used as a technique to measure masses of proteins/peptides in complex mixtures obtained from biological samples. This provides tremendous potential to study disease proteome and to identify drug targets directly at the protein/peptide level [1].

In a typical proteomic experiment, a huge volume (e.g. 1 GB) of MS data is often generated. Each of MS spectra consists of two large vectors corresponding to mass to charge ratio (*m*/*z*) and intensity value, respectively. The first step in proteomic data analysis is to extract peptide induced signals (i.e., peaks) from raw MS spectra. Peak detection is not only a feature extraction step, but also an indispensable step for subsequent protein identification, quantification and discovery of disease-related biomarkers [2,3]. However, peak detection is a challenging task since mass spectra are often corrupted by noise. As a result, various algorithms have been proposed to facilitate the identification of informative peaks that correspond to true peptide signals. These algorithms differ from each other in their principles, implementations and performance. In order to provide a comprehensive comparison of existing peak detection algorithms and extract reasonable criteria for developing new peak detection methods, we need to answer the following questions:

1. What's the working mechanism of an algorithm?

2. What are the differences and common points among different algorithms?

3. What is their performance in MS data analysis?

To address the above questions, we study the peak detection process using a common framework: smoothing, baseline correction and peak finding. Such a decomposition enables us to better elucidate the fundamental principles underlying different peak detection algorithms. More importantly, it helps us to clearly identify the differences and similarities among existing peak detection algorithms.

We describe each part in the peak detection process with particular emphasis on their technical details, hoping that this can help readers implement their own peak detection algorithms.

During evaluation, we choose five typical peak detection algorithms to conduct a comparative experimental study. In the experiments, we use both simulation data and real MALDI MS data for performance comparison. The results show that the continuous wavelet-based algorithm provides the best average performance.

The remainder of this paper is organized as follows: section 2 provides details on existing peak detection algorithms and highlights their differences and similarities; section 3 conducts a performance comparison on some typical peak detection algorithms using simulation data and real MALDI MS data; section 4 concludes the paper.

## Methods

### Peak Detection Process

Usually, peptide signals appear as local maxima (i.e., peaks) in MS spectra. However, detecting these signals still remains challenging due to the following reasons:

(1) Some peptides with low abundance may be buried by noise, causing high false positive rate of peak detection.

(2) The chemical, ionization, and electronic noise often result in a decreasing curve in the background of MALDI/SELDI MS data, which is referred to as baseline [4]. The existence of baseline produces strong bias in peak detection. It is desirable to remove baseline before peak detection.

**Figure 1:**
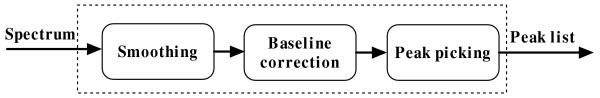
**Peak detection framework**. The input mass spectrum is transformed into a list of peaks.

**Figure 2:**
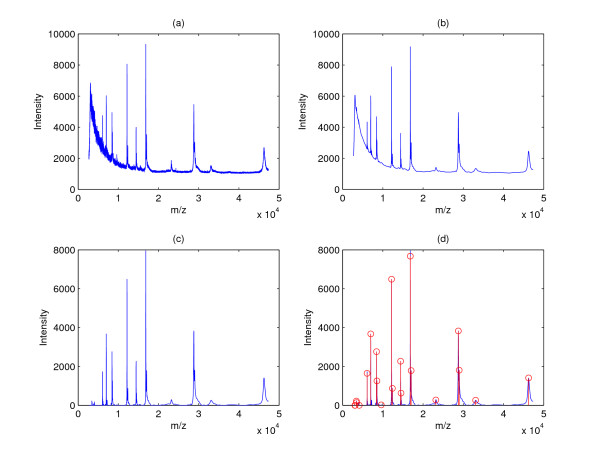
**An example of the peak detection process**. (a) A raw spectrum, (b) the spectrum after smoothing, (c) the spectrum after smoothing and baseline correction and (d) final peak detection results with peaks marked as circles.

To facilitate peak detection, we often use the framework shown in Figure 1. It should be noted that smoothing and baseline correction may switch their locations in the pipeline. Figure 2 gives a concrete example of peak detection by showing the result after each step of the pipeline.

### Categorization

Existing peak detection algorithms can be categorized according to the methods used in each step of peak detection process. Table 1 lists some popular MS data analysis methods and their peak detection functions. In this paper, we use CWT to denote MassSpecWavelet and LMS to denote Local Maximum Search. Similarities and differences among these methods can be addressed from the table. Here we would like to highlight the following:

**Table 1:** Open source software packages for MS data analysis

**Program**	**S**	**B**	**P**	**Web link**
Cromwell [12]	S7	B1	P1, P4	

LCMS-2D [20]	-	B5	P1, P2	

LIMPIC [21]	S4	B2	P1, P3	

LMS [22]	S3	B2	P1, P4	

MapQuant [16]	S1,S2,S3	-	P7	

CWT [10]	S5	B4	P1, P6	

msInspect [23]	S6	B2	P5	

mzMine [24]	S1, S2	-	P1, P2, P8	

OpenMS [15]	S5	B4	P7	

PROcess [13]	S1	B2, B3	P1, P2, P5	

PreMS [25]	S7	B1	P1, P4	

XCMS [8]	S3	-	P1, P4	

Here "**S**" denotes smoothing filter, "**B**" denotes baseline correction method, "**P**" denotes peak finding criterion and "-" means smoothing or baseline correction method is not used. Cromwell, LIMPIC, LMS, CWT, and PROcess are designed for single spectrum peak detection. LCMS-2D, MapQuant, msInspect, mzMine, OpenMS and XCMS are designed for LC-MS (Liquid Chromatography Mass Spectrometry) data analysis. PreMS is a GUI (Graphic User Interface) package based on Cromwell.

(1) The algorithms in Table 1 are chosen according to three criteria:

• The software is mainly designed for MS data pre-processing.

• The software is open source.

• The software is described in a publication.

(2) In Table 1, S1-S7, B1-B5 and P1-P8 denote different smoothing methods, baseline correction methods and peak finding criteria, respectively. We shall provide their details in subsequent sub-sections.

• Smoothing

S1: Moving average filter

S2: Savitzky-Golay filter

S3: Gaussian filter

S4: Kaiser window

S5: Continuous Wavelet Transform

S6: Discrete Wavelet Transform

S7: Undecimated Discrete Wavelet Transform

• Baseline Correction

B1: Monotone minimum

B2: Linear interpolation

B3: Loess

B4: Continuous Wavelet Transform

B5: Moving average of minima

• Peak Finding Criterion

P1: SNR

P2: Detection/Intensity threshold

P3: Slopes of peaks

P4: Local maximum

P5: Shape ratio

P6: Ridge lines

P7: Model-based criterion

P8: Peak width

### Smoothing Filters

These methods usually apply traditional signal processing techniques such as moving average filter, Savitzky-Golay filter and Gaussian filter. For an input spectrum, we represent it as [*m*/*z*, *x*] with the first element as *m*/*z *vector and the second element as intensity vector (with equal length). To facilitate descriptions in signal processing, we further use *x*(*t*) to denote the continuous form of intensity vector and use *x*[*n*] to denote the discrete form of intensity vector. Here *t *and *n *serve as indexing variables. The input spectrum is always discrete. We use the continuous form to be consistent with the original description. In real applications, we usually sample the continuous filter to obtain its discrete form. We can obtain *m*/*z *values from *m*/*z *vector easily by using the corresponding indexing variable as well. A spectrum after smoothing can be expressed as *y*[*n*] = *x*[*n*] * *w*[*n*] for discrete case and *y*(*t*) = *x*(*t*) * *w*(*t*) for continuous case, where * denotes convolution operation. In above equations, *w*[*n*] and *w*(*t*) are a weight vector and a weight function, respectively. The use of different *w*[*n*] and *w*(*t*) will lead to different filters.

S1: **Moving average filter **[5]:

The output of the moving average filter *y*[*n*] reads:

(1) $$y[n]=x[n]*w[n]=\frac{1}{2k+1}\sum_{i=-k}^{k}x[n-i],$$

where $w[n]=\frac{1}{2k+1}$, -*k *≤ *n *≤ *k*. The odd number 2*k *+ 1 denotes filter width. The greater the filter width, the more intense the smoothing effect.

S2: **Savitzky-Golay filter**:

The Saviztky-Golay filtering can be considered as a generalized moving average filter. It performs a least squares fit of a small set of consecutive data points to a polynomial and takes the central point of the fitted polynomial curve as output.

**Figure 3:**
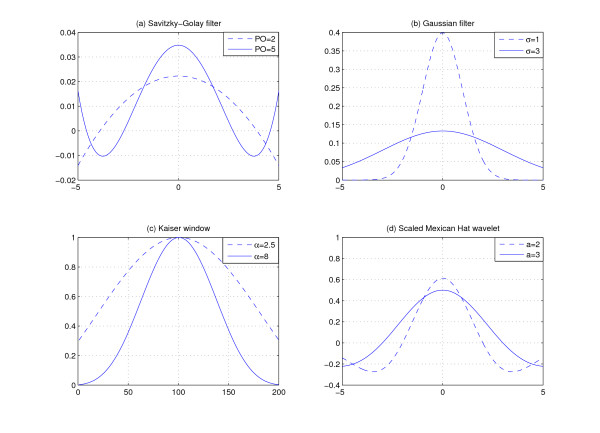
**Smoothing filters**. In (a), "PO" stands for polynomial order of polynomial fitting in Savitzky-Golay filter. In (b), *σ *is the standard deviation. In (c), *α *determines the shape of Kaiser window. In (d), *a *is the scale of the wavelet.

The smoothed data point *y*[*n*] after Savitzky-Golay filtering is given by the following equation:

(2) $$y[n]=x[n]*w[n]=\frac{\sum_{i=-k}^{k}A_{i}x[n-i]}{\sum_{i=-k}^{k}A_{i}},$$

where $w[n]=\frac{A_{n}}{\sum_{i=-k}^{k}A_{i}}$, -*k *≤ *n *≤ *k*. Here, *A*_*i *_controls the polynomial order. Figure 3(a) shows Savitzky-Golay filters with different polynomial orders. For more information about *A*_*i*_, please refer to [6].

S3: **Gaussian filter**

After a signal *x*(*t*) passing Gaussian filter, the output reads:

(3) $$y(t)=x(t)*w(t)=\int_{-\infty }^{+\infty }x(\tau )w(t-\tau )d\tau ,$$

where $w(t)=\frac{1}{\sqrt{2\pi }\sigma }e^{-\frac{t^{2}}{2\sigma^{2}}}$. The degree of smoothing is determined by the standard deviation *σ*. In fact, we can view Gaussian filter as a weighted moving average filter. This filter sets larger weight factors for points in the center and smaller weight factors for points away from the center. Figure 3(b) shows Gaussian filters with different *σ*.

Some researchers use the second-derivative of Gaussian to perform smoothing. Their argument is that the second-derivative of Gaussian can implicitly remove background when smoothing signals [7,8].

S4: **Kaiser window**

After a signal passing a Kaiser window:

(4) $$y[n]=x[n]*w[n]=\sum_{i=-\infty }^{+\infty }x[i]w[n-i],$$

where $w[n]=\frac{I_{0}(\alpha \sqrt{1-{\left(\frac{2n}{N}-1\right)}^{2})}}{I_{0}(\alpha )}$, 0 ≤ *n *≤ *N*. *α *determines the shape of the Kaiser window. A large *α *indicates a sharp Kaiser window. *N *denotes the width of window. *I*_0 _is zeroth-order modified Bessel function of the first kind [9]. Figure 3(c) shows two Kaiser windows with different *α *values.

S5, S6, S7: **Wavelet based filters**

Wavelet can be grouped as continuous wavelet transform and discrete wavelet transform. The continuous wavelet transform can be written as

(5) $$y(t)=x(t)*w(t)=\frac{1}{\sqrt{\left|a\right|}}\int_{-\infty }^{+\infty }x(\tau )\psi (\frac{t-\tau }{a})d\tau ,$$

where $w(t)=\frac{1}{\sqrt{\left|a\right|}}\psi (\frac{t}{a})$. a denotes scale and *ψ *denotes mother wavelet function. In continuous wavelet analysis, Du *et al *[10]choose Mexican Hat wavelet. Mexican Hat wavelet reads as:

(6) $$\psi (t)=\frac{2}{\sqrt{3}\pi^{1/4}}(1-t^{2})e^{-t^{2}/2}.$$

**Figure 4:**
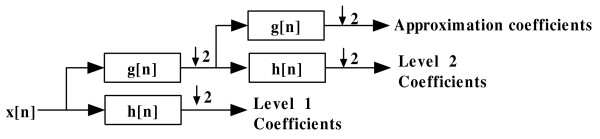
**The process of computing DWT**. Here "↓ 2" means down sampling by 2, *h*[*n*] is a high-pass filter and *g*[*n*] is a low-pass filter.

Then *w*(*t*) forms a scaled Mexican Hat wavelet. Figure 3(d) shows *w*(*t*) with different *a*. Here, *a *determines the width of the wavelet. With different *a*, we can use *w*(*t*) to model peaks with different width. This is especially important for low-resolution data in which peak width varies a lot. Peaks with higher *m*/*z *values tend to have larger width. Using fixed-window filters will not perform well in this case. Discrete wavelet transform computes on scales and translations based on the power of two. Figure 4 shows a typical method for computing discrete wavelet transform, where *h*[*n*] is a high-pass filter and *g*[*n*] is a low-pass filter. The procedure to compute discrete wavelet transform is as follows:

(1) Signal is decomposed simultaneously by a low-pass filter *g*[*n*] and a high-pass filter *h*[*n*].

(2) The output of *h*[*n*] is then down sampled by two to generate detail coefficients and the output of *g*[*n*] is down sampled by two to generate approximation coefficients. The coefficients obtained from the output of *h*[*n*] are named level one coefficients.

(3) The output of *g*[*n*] goes through another group of high-pass filter and low-pass filter. Steps (1) and (2) go on until we obtain the last level of coefficients.

The advantage of discrete wavelet transform over continuous wavelet transform is its efficiency because it only computes on the scales and positions based on the power of two, while the redundancy of continuous wavelet transform makes the interpretation of MS peak detection easier [10].

Discrete wavelet transform is shift-variant. To achieve shift invariance, undecimated discrete wavelet transform has been proposed [11,12].

### Baseline Correction

Baseline correction is typically a two-step process: (1) estimating the baseline and (2) subtracting the baseline from the signal. In the following, we list details of some commonly used baseline correction methods. Since baseline substraction is straightforward, we mainly focus on the baseline estimation procedure in different methods.

B1: **Monotone minimum**

This method includes two steps to estimate baseline. The first step is to compute the difference, which can be used to determine the slope of each point. Then, this method starts from the leftmost point *A *in the spectrum and continues the following procedure until the rightmost point is reached:

• If the slope of a local point *A *is smaller than zero, a nearest point *B *to the right of *A *whose slope is larger than zero is located. All points between *A *and *B *serve as baseline between *A *and *B*.

• If the slope of a local point *A *is larger than zero, a nearest points *B *to the right of *A *whose intensity is smaller than *A *is located. The intensity of every point on the result baseline between *A *and *B *equals to the intensity of *A*.

• Let *A *= *B*.

B2: **Linear interpolation**

Linear interpolation takes two steps to estimate baseline:

• Divide the raw spectrum into small segments and use the mean, the minimum or the median of the points in each segment as the baseline point.

• Generate a baseline for the raw spectrum by linearly interpolating baseline points across all small segments.

B3: **Loess**

First, it divides the raw spectrum into small segments. Then, in each small segment, it computes the quantile. After that, it estimates a predictor in every small segment for baseline estimation. The predictor in each small segment is obtained using the following rules:

• If the intensity of a point *A *is smaller than the quantile in the segment, then the intensity of corresponding point on predictor equals the intensity of *A*.

• If the intensity of a point is larger than or equal to the quantile in the segment, then the intensity of corresponding point on predictor equals the quantile.

Baseline is obtained by applying local polynomial regression fitting to the predictor.

B4: **Continuous Wavelet Transform**

In local regions, baselines are monotonic. Baseline can be modeled as the following function:

(7) *Base *= *B*(*t*) + *C*,

where *C *is a constant and *B*(*t*) is an odd function [10]. The continuous wavelet transform of the equation reads:

(8) *Base*(*a*, *b*) = ∫ *B*(*t*)*ψ*_*a*,*b*_(*t*)*dt *+ ∫ *Cψ*_*a*,*b*_(*t*)*dt*,

where $\psi_{a,b}(t)=\frac{1}{\sqrt{\left|a\right|}}\psi (\frac{t-b}{a})$. Because wavelet function has zero mean, the second term of equation (8) is

zero. If we use a symmetric wavelet function (like Mexican Hat wavelet), the first item in Equation (8) is also zero. Thus, continuous wavelet transform removes baseline automatically.

B5: **Moving average of minima**

This method uses two steps to estimate baseline:

• Estimate a rough baseline by finding local minimum within a two Da window for each point.

• Use a moving window to smooth the rough baseline obtained in the first step.

### Peak Finding Criteria

There are many peak detection methods. Most methods detect peaks after smoothing and baseline correction. However, it should be noted that there is a special case, CWT does not have explicit smoothing and baseline correction steps. Du *et al*. [10] claim that baseline can be removed if continuous wavelet transform is carried out on a raw spectrum. We have shown this fact in Section Baseline Correction. In the following, we illustrate the criteria used by different algorithms to find similarities among different algorithms.

P1: **SNR**

SNR stands for signal to noise ratio. Different methods define noise differently. Below are two examples:

• Noise is estimated as 95-percentage quantile of absolute continuous wavelet transform (CWT) coefficients of scale one within a local window [10].

• Noise is estimated as the median of the absolute deviation (MAD) of points within a window [13].

P2: **Detection/Intensity threshold**

This threshold is used to filter out small peaks in flat regions. In these regions, the median of the absolute deviation (MAD) is quite small, which may result in big SNR. Using SNR alone may identify many noisy points as peaks.

P3: **Slopes of peaks**

This criterion uses the shape of peaks to remove false peak candidates. In order to compute the left slope and the right slope of a peak, both the left end point and the right end point of the peak need to be identified. Peak candidate is discarded if both left slope and right slope are less than a threshold. The threshold is defined as half of the local noise level [14].

P4: **Local maximum**

A peak is a local maximum of *N *neighboring points.

P5: **Shape ratio**

Peak area is computed as the area under the curve within a small distance of a peak candidate. Shape ratio is computed as the peak area divided by the maximum of all peak areas. The shape ratio of a peak must be larger than a threshold.

P6: **Ridge lines**

Ridge lines are obtained in the following steps:

• Carry out continuous wavelet transform on raw spectrum. This step produces 2-D coefficient matrix with size of *M *× *N*, where *M *is the number of scales and *N *is the length of spectrum.

• Connect nearest local maximal coefficients of adjacent scales to obtain ridge lines. The distance between two adjacent points on a ridge line should be smaller than a window size.

• Use a variable *gap *to count how many successive times that a local maximal coefficient can not find its nearest counterpart in the next scale. If the *gap *is larger than a given threshold, the ridge line is dropped.

Ridge lines are used in the following ways:

• False peaks are removed if the length of their ridge lines are smaller than a given threshold supplied by users.

• The width of a peak is proportional to the scale corresponding to the maximum amplitude on the ridge line [10]. A peak candidate is dropped if its width is not in a given range.

P7: **Model-based criterion**

The application of this criterion consists of three steps:

• Locate the endpoints of both sides for each peak. The left endpoint and right endpoint of a peak define its peak area.

• Estimate the centroid for each peak. For *m*/*z *axis, the centroid of a peak is computed as intensity-weighted average of points within the peak area [15].

• Use a model function to fit peaks.

Different methods choose different model functions to fit peaks. OpenMS [15] chooses asymmetric Lorentzian or sech^2 ^function while MapQuant [16] uses Gaussian function to fit peaks.

P8: **Peak width**

The two end points of a peak define its peak area. The intensities of all points within the peak area should be larger than a given noise level. A simple way to locate a peak area is to start from a point with intensity above a given noise level and move to the right until we run into a point with intensity below the noise level.

After peak end points have been identified, peak width is computed as the mass difference of right end point and left end point. The peak width should be within a given range.

## Results and discussion

### Data Description and Algorithm Selection

In comparison, we use one group of simulation data and one group of real MALDI MS data. The low resolution simulation data is downloaded from the website of M. D. Anderson Cancer Center [17,18]. The high resolution real data is obtained from Aurum Data Set [19], which contains known purified and tryptic-digested proteins. For simulation data, the number of true peaks in a spectrum is around 70 on average. The *m*/*z *range is between 400 Da and 64800 Da. The mass variation is: Δ*m *∈ [0.251 *Da*, 3.915 *Da*]. The median of SNR is around 0.675. For real data, the number of true peaks in a spectrum varies from 50 and 100. The *m*/*z *range is between 800 Da and 3500 Da. The mass variation is: Δ*m *∈ [0.016 *Da*, 0.034 *Da*]. The median of SNR is around 4.854. The reader is referred to additional file 1 for more details on the data.

Software programs for LC-MS data analysis consider additional information along the LC-axis during peak detection. In order to obtain a fair comparison, here we only focus on single spectrum based peak detection algorithms. According to this criterion, only five algorithms in Table 1 will remain: Cromwell, CWT, LMS, LIMPIC and PROcess. These algorithms are designed to analyze MALDI MS data. They can also be used to analyze MS/MS data in a spectrum by spectrum manner. It should be noted that these methods are very representative as LC-MS oriented programs also use similar ideas for peak detection along the *m*/*z *axis.

### Evaluation Criteria

In simulation data, the list of ground-truth peaks is the input before data generation. In real data, the trypsin-digested theoretical peaks (without adding isotope masses) are used as the ground-truth peaks. In both cases, a detected peak is labeled as a false peak if its mass is not within the ± 1% error range of the expected *m*/*z *value. Multiple peaks within the error range will be considered as one peak. We use false discovery rate (FDR) and sensitivity to measure the performance of algorithms. False discovery rate is defined as the number of falsely identified peaks divided by the total number of peaks found by algorithms. Sensitivity is defined as the number of correctly identified peaks divided by the total number of true peaks. For two algorithms with the same false discovery rate, the larger the sensitivity, the better the algorithm performance.

It is difficult for two algorithms to produce the same false discovery rate. Here we divide false discovery rate into small segments. Such segments have clear interpretations. For example, the FDR [0,0.1] range reveals the algorithm's ability to recognize the most abundant (based on SNR) peaks in the spectrum. Every time when we obtain peak lists, both false discovery rates and sensitivity are computed. We group the sensitivity together if the corresponding false discovery rates fall into the same small segment. Then average values of sensitivity in the same group are computed. The average value of sensitivity is used to evaluate the performance of one algorithm in that area.

As ground truth is known for both simulation data and real data in this paper, the ROC curve is probably the most informative measure for evaluation of different peak detection methods. However, the false discovery rates of wavelet-based methods are limited to a relatively small range across all possible parameter settings. On one hand, this reflects the robustness of wavelet-based methods. On the other hand, the plot of ROC curve becomes difficult in wavelet-based methods. Here we use the following alternative method to conduct performance comparison: we select four regions of false discovery rate:[0, 0.1), [0.2, 0.3), [0.4, 0.5), [0.6, 0.7) and compare sensitivity of different algorithms in these regions using boxplot. Such strategy is capable of providing an overall performance evaluation since it is roughly a "discrete" ROC curve in four regions. Moreover, the boxplots illustrate the performance variances of different algorithms.

Different programs have different parameters to adjust when performing peak detection. Since it is very time consuming to optimize each algorithm using all potential combinations of different parameters, we mainly test combinations of parameters that are related to peak finding and use default values for other parameters. Please refer to additional file 2 for more details.

### Comparison of Algorithms Using Simulation Data

The simulation data is generated using a model that incorporates some characteristics of real MALDI-TOF mass spectrometers: The simulation engine takes a peak list with both *m*/*z *values and intensity values as input and generates an artificial spectrum as output. The user-specified peaks are labeled as the ground-truth during data generation, while other peaks are labeled as false peaks in the simulation spectrum. In addition, the simulation engine assumes that the isotopic distribution follows the Bernoulli distribution. It also includes exponential baseline curve and Gaussian additive noise.

This data set has 25 groups of data and each group has 100 spectra. Each spectrum has a true peak list provided by data set. We directly use these peak lists as ground truth in our experiment. We use different parameter settings to perform peak detection repeatedly on 100 spectra in the same group, and then compute the average value of sensitivity with corresponding false discovery rates locating in the same small region. For each algorithm, we obtain 25 average values of sensitivity in each small region.

**Figure 5:**
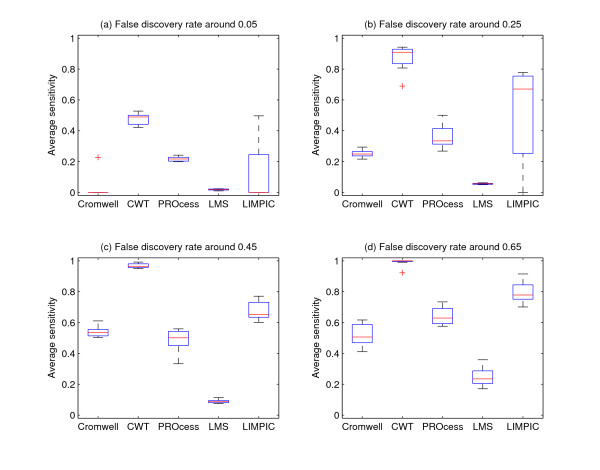
**Performance of different algorithms at different false discovery rates using simulation data**. In this figure, (a), (b), (c) and (d) show the average sensitivity when false discovery rate is around 0.05, 0.25, 0.45 and 0.65, respectively.

Figure 5 shows the performance of five algorithms. CWT provides the best performance among these algorithms. Our explanation is that the use of wavelets in baseline modeling/correction and the use of ridge lines enable CWT-based algorithm to achieve better performance.

### Comparison of Algorithms Using Aurum Data

Aurum Dataset is a high resolution data set, which contains spectra from 246 known, individually purified and trypsin-digested protein samples with an ABI 4700 MALDI TOF/TOF mass spectrometer. In the experiments, we do not use MS/MS data and limit our analysis only to MS spectra. For each MS spectrum, we generate the ground truth peaks in silico using the following parameters: trypsin digestion with a maximum of one missed cleavage, monoisotopic peaks and single charge state. We also consider some typical PTMs (Post-Translational Modifications): carboxyamidomethyl cysteine as the fixed modification and oxidation of methionine as the variable modification. Note that peptides having missed cleavages and PTMs are also used to generate ground-truth peaks. After obtaining the theoretic peak list, we merge identical peaks into one peak and delete peaks whose *m*/*z *values are not in the range between 800 Da and 3500 Da. We select 200 spectra, and divide the spectra into eight groups. We perform the same performance test as we did for the simulation data.

**Figure 6:**
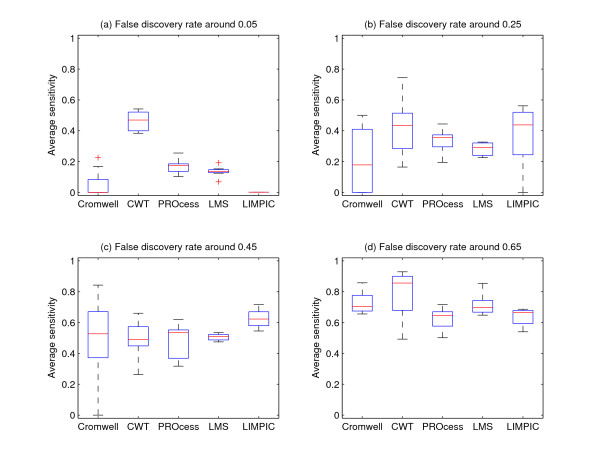
**Performance of different algorithms at different false discovery rates using Aurum data**. In this figure, (a), (b), (c) and (d) show the average sensitivity when false discovery rate is around 0.05, 0.25, 0.45 and 0.65, respectively.

Figure 6 shows the performance of these five methods. The noisy nature of real data causes larger performance variations of most methods in this experiment. LIMPIC achieves comparable performance as CWT in Figure 6(b) and outperforms CWT in Figure 6(c). On average, CWT provides the best results.

### Report of Running Time

For high-throughput data analysis, high efficiency is always desirable. In Table 2, we list the average running time of different algorithms on both simulation data and Aurum data, respectively. We obtain the running time of different algorithms using their original software packages on the same PC. It should be noted that these programs are implemented in different languages. Even without considering the implementation efficiency, complexity comparison is reasonable only for programs implemented in the same language. In this sense, Table 2 only serves as a reference for those readers who are interested in computational cost.

**Table 2:** Average processing time per spectrum using different programs

**Program**	**Platform**	**Time for Simulation data (Second)**	**Time for Real Data (Second)**
Cromwell	Matlab	0.21	1.71

LMS	Matlab	0.50	3.23

LIMPIC	Matlab	1.74	1.59

CWT	R	3.31	11.00

PROcess	R	4.56	33.21

### Parameter Tuning

When the false discovery rate is 5%, half of true peaks are not detected; when 90% of true peaks are detected, many other identified peaks are noise peaks. We use the *F*1 measure to measure the performance of an algorithm by compromising between false discovery rate and sensitivity. The *F*1 measure is defined as:

(9) $$F1=\frac{2\times (1-FDR)\times Sensitivity}{1-FDR+Sensitivity}.$$

The larger *F*1 is, the better a parameter combination will be. We exhaustively try combination of parameters and count the numbers that a parameter combination provides the maximal *F*1. The parameter combination that produces the largest number of maximal *F*1 is considered as the best combination. We also test the peak detection precision for each algorithm with its best parameter combination. For readers who are interested in parameter settings, please refer to the additional file 2 for more information.

## Conclusion

In this paper, we provide a comprehensive survey of existing peak detection methods. In addition, we compare performance of five single spectrum based peak detection algorithms. Results show that CWT provides the best performance.

The reasons that CWT provides the best performance are two-fold:

(1) CWT optimally characterizes the shape of peaks in mass spectra. In a real spectrum, peak width varies a lot [10]. Hence smoothing the spectrum using fixed-window filters may fail. CWT avoids the problem by performing multi-scale smoothing.

(2) True peptide-related peaks are more consistent at multiple scales than false positive peaks that are mainly caused by high frequency noise. The concept of forming ridge lines in CWT effiectively removes false positive peaks.

Algorithms studied in this paper mainly focus on how to identify peak positions correctly. They ignore how to compute peak abundance, which is very important in some applications (e.g. protein quantification). In our future work, we plan to study the issue of peak detection in LC-MS data. It will be interesting to see if additional information along the LC-axis may help to improve peak detection results.

## Authors' contributions

CY performed the implementations and drafted the manuscript. ZH participated in the categorization of related work. WY conceived the study and finalized the manuscript. All authors read and approved the final manuscript.

## Supplementary Material

Additional file 1 **Data and results**. This file lists the data used in this paper and the results for the experiments. Click here for file

Additional file 2 **Parameter setting**. This file gives parameters settings in experiments for each program compared in this work. Click here for file
