# Supplementary material for: Comparison of public peak detection algorithms for MALDI mass spectrometry data analysis
Source: BMC Bioinformatics. 2009 Jan 6;10:4. doi: 10.1186/1471-2105-10-4 (PMC2631518; doi:10.1186/1471-2105-10-4)
Supplement: Additional file 1 — Data and results. This file lists the data used in this paper and the results for the experiments. [file 1471-2105-10-4-S1.doc]

**Comparison of public peak detection algorithms for MALDI mass spectrometry data analysis**

Supplementary documents for the manuscript.

(1)All the programs used in comparison are open source and can be downloaded from the following websites:

- [Cromwell](http://bioinformatics.mdanderson.org/software.html): Anderson Cancer Center
- [LMS](http://bioinformatics.ust.hk/LMS.zip)
- [MassSpecWavelet](http://www.bioconductor.org/packages/devel/bioc/html/MassSpecWavelet.html) : Bioconductor packages
- [PROcess](http://www.bioconductor.org/packages/devel/bioc/html/PROcess.html) : Bioconductor packages
- [LIMPIC](http://www.biomedcentral.com/1471-2105/8/101/additional/)

(2)Data we use

- [Simulation data](http://bioinformatics.mdanderson.org/Supplements/Datasets/Simulations/index.html): Anderson Cancer Center, we select the first 25 data sets.
- [Real data](http://www.proteomecommons.org/archive/1122567790437/index.html): Aurum Data Set. We select 200 spectra and generate ground truth for them.

1.[Raw data](ftp://143.89.46.202/RawSpectrum.7z)(7z file)

2.[True peaks](ftp://143.89.46.202/TruePeaks.7z)(7z file)

(3)Results:

1.[Results for simulation data](http://bioinformatics.ust.hk/Simulate.zip) (zip file)

2.[Results for real data](http://bioinformatics.ust.hk/Real.zip) (zip file)
