# Supplementary material for: Comparison of public peak detection algorithms for MALDI mass spectrometry data analysis
Source: BMC Bioinformatics. 2009 Jan 6;10:4. doi: 10.1186/1471-2105-10-4 (PMC2631518; doi:10.1186/1471-2105-10-4)
Supplement: Additional file 2 — Parameter setting. This file gives parameters settings in experiments for each program compared in this work. [file 1471-2105-10-4-S2.doc]

Simulation data set has 25 groups of data. Each group consists of 100 spectra. Real data has 8 groups of data, Each
group consists of 25 spectra. Please refer to <http://bioinformatics.ust.hk/MSPeakComparison.html> for more information.

**Part one: parameters**

| Program Name | Parameters Names In Package |
| --- | --- |
| CWT | peakScaleRange,snr and amp.Th |
| PROcess | snr,area and ratio |
| LIMPIC | factor, mz_block, iupac_thres and peak_width |
| LMS | neighbor_size and scale_thres |
| Cromwell | waveletThreshold |

**Part two: parameter values**

*MassSpecWavelet parameters:
peakScaleRange=[2, 4, 6];
snr=[1, 3, 5];
amp.Th=[0.0001, 0.01, 0.1]

*PROcess parameters:
snr=[1, 3, 5];
area=[0.3, 0.003, 0.0003];
ratio=[0.001, 0.01, 0.1, 0.5]

*LIMPIC parameters:
factor=[10, 20, 30, 40];
mz_block=[50, 100, 150, 200];
iupac_thres=[3, 5, 7];
peak_width=[0.5, 1, 2];

*Local maximum search:
When data resolution is low (e.g. When most intervals between two adjacent points are around 1Da to 3Da), the neighborhood is set as around 15Da. the neighbor_size in the unit of points is determined as 15/1 = 15 points, scale_thres is set as 5.
In experiment:
neighbor_size = [1, 4, 8, 12, 16, 20, 26, 30];
scale_thres = [1, 3, 5, 7, 9]

When data resolution is high (e.g. When most intervals between two adjacent points are around 0.02Da), the same neighbor_size of 15Da will result in different number of points: 15/0.02=750 points
In experiment:
neighbor_size=[70, 100, 150, 300, 450, 600, 850, 1240]
scale_thres=[8, 12, 16, 10, 24]

*Cromwell
Similar argument as in *Local maximum search
When data resolution is low, wavelet threshold is set as around 15,
In experiment:
waveletThreshold=4:2:30

When data resolution is high, wavelet threshold is set as around 750,
In experiment:
waveletThreshold=[60, 100, 300, 400, 600, 700, 900, 1000, 1200, 1400, 1800];

**Part three: parameters for obtaining the best F1 measure**

In our paper, we use a method similar to ROC curve to measure the overall performance for each algorithm. And we use F1 measure to obtain a best compromise between false discovery rate and sensitivity. We also test peak detection precision for each algorithm with its best parameter combination.

|  | Using Simulation Data | | | Using Real Data | | |
| --- | --- | --- | --- | --- | --- | --- |
| Program Name | Parameter Values | The number of times to generate maximal F1  (Total number is 2500) | Median m/z error | Parameter Values | The number of times to generate maximal F1  (Total number is 200) | Median m/z error |
| CWT | [peakScaleRange,snr,amp.Th]=  [2,1,0.01] | 1068 | 0.32% | [peakScaleRange,snr,amp.Th]=[2 1 0.1] | 148 | 0.17% |
| PROcess | [snr,area,ratio]=[1,0.003,0.01] | 632 | 0.35% | [snr,area,ratio]=[1,0.0003,0.1] | 93 | 0.19% |
| LIMPIC | [factor,mz_block,iupac_thres,peak_width]= [40,50,3,0.5] | 306 | 0.40% | [factor,mz_block,iupac_thres,peak_width]= [10,50,7,0.5] | 117 | 0.59% |
| LMS | [neighbor_size,scale_thres] = [8,5] | 1471 | 0.52% | [neighbor_size,scale_thres] = [300,8] | 31 | 0.53% |
| Cromwell | waveletThreshold=30 | 2050 | 0.40% | waveletThreshold=1800 | 81 | 0.34% |
